# Caught on Camera: On the Need of Responsible Use of Video Observation for Animal Behavior and Welfare Research

**DOI:** 10.3389/fvets.2022.864677

**Published:** 2022-04-25

**Authors:** Mona F. Giersberg, Franck L. B. Meijboom

**Affiliations:** Animals in Science and Society, Department Population Health Sciences, Faculty of Veterinary Medicine, Utrecht University, Utrecht, Netherlands

**Keywords:** animal study, video, privacy, research integrity, co-creation, participatory approach

## Abstract

Video analysis is a popular and frequently used tool in animal behavior and welfare research. In addition to the actual object of research, video recordings often provide unforeseen information about the progress of the study, the animals or the people involved. Conflicts can arise when this information is weighed against the original intention of the recordings and broader social expectations. Uncertainty may prevent the video observers, often less experienced researchers, to properly address these conflicts, which can pose a threat to animal welfare and research quality and integrity. In this article, we aim to raise awareness of the interrelationship of variables characteristic for video-based animal studies and the potential conflicts emerging from this. We propose stepping stones for a framework which enables a culture of openness in dealing with unexpected and unintended events observed during video analysis. As a basis, a frame of reference regarding privacy and duty of care toward animals should be created and shared with all persons involved. At this stage, expectations and responsibilities need to be made explicit. During running and reporting of the study, the risk of animal welfare and research integrity issues can be mitigated by making conflicts discussible and offering realistic opportunities on how to deal with them. A practice which is outlined and guided by conversation will prevent a mere compliance-based approach centered on checklists and decision trees. Based on these stepping stones, educational material can be produced to foster reflection, co-creation and application of ethical practice.

## Introduction

Video observation is a popular tool for behavior research in animals. A main advantage of video observations is that the animals are not influenced by the presence of an observer (1). Depending on the type of camera, it is also possible to monitor animals in areas which are difficult to access, such as nest boxes in laying hen husbandry, and during night-time (2, 3). Further advantages of video observation include the possibility of archiving and sharing of raw footage, which supports the principles of Open Science (4).

However, video recordings sometimes provide unforeseen information about the study, the people or the animals involved. The most obvious example may be the unintentional recording of a caretaker who unexpectedly mistreats the study animals. However, also human carelessness, such as leaving the lights on during night, private conversations between animal caretakers, or technical defects, such as skipping a feeding time with automatic feeders, can be unintentionally documented. These events provide scope for conflict, which emerges from the interactions of the variables outlined hereafter. First, there is the object or what is seen on the video, which we define as an action, an omission or more neutrally as an event. This object involves the observed, who may be the study animal. However, the observed may also be a human who is captured on the video while caring for the animal or carrying out routine work in the barn. The object and the observed are seen by the one who watches the videos, the observer. The appearance of the object and the observed on the video can be expected or unexpected, and intended or unintended. Further dimensions to be considered are the place and the time in which the object and the observed are filmed on the one hand, and in which the videos are watched on the other.

Once recognized, the event may cause uncertainty in the observer. How should they deal with it? Does the welfare of the animals outweigh the privacy of the caretaker? Can the study still be published if it is admitted that the lights were on unintentionally? Is the situation observed a problem at all? To deal with these events, the general principles of privacy and research integrity are an essential starting point (5, 6). As a precondition, people involved in a video-based animal study need to be aware of their institutions' policies (or the absence of such), for instance on consent or data confidentiality. However, these principles are not sufficient to address the situations' full complexity, which arises from the fact that animals are involved. Their dependency on human care and lack of self-reporting capacities leaves them in a vulnerable position. At the same time people have more or less agreed on the moral status of animals, which leads to human obligations in terms of animal welfare and a duty of care (7).

Existing frameworks and guidelines for animal behavior research mainly focus on methodology. The literature ranges from general advice (1) to articles on specific topics, for instance on choosing accurate video sampling strategies in poultry research (8). Although a more recent edition of the standard work “Measuring behavior” (9) discusses animal and human research ethics, “dubious research practices”, and responsibility and integrity when publishing results, there still are no approaches addressing the specific conflicts that can arise during video analysis as outlined above. Adopting frameworks from other disciplines that deal with similar issues does not seem to be a viable option either. In social science, video recordings are broadly used as investigative tools. However, comparable to animal science, there seems to be a lack of theoretical reflection on these methods and on the potential ethical conflicts and uncertainties arising from them in practice (10, 11). This particularly applies to situations in which unexpected and unintended information is obtained on a study participant's activities which are considered as “illegal, amoral, immoral or otherwise illicit” (11). Although the ethical complexity of such situations is recognized, there seem to be no frameworks to assist social scientists in dealing with them, for instance by identifying and evaluating the unforeseen information, determining the people involved and deciding whether or not it is required to report this information to any authority (11).

The overall aim of this perspective article is to raise awareness of the potential conflicts and dilemmas emerging from the interrelationship of the variables outlined above in the context of video analysis in animal behavior and welfare research. First, we argue that in this specific context, the conflicts cannot be addressed by mere reference to more general issues of privacy and research integrity. The fact that animals are involved further complicates the picture. Therefore, we propose stepping stones for a framework which aims to enable open discussion about unexpected and unintended events observed during video analysis, clarification of privacy issues, and translation of existing approaches regarding research integrity to the specific context of video analysis in animal research. Based on this framework, educational material can be produced to foster the co-creation and application of ethical practice involving all persons engaged in a video-based animal study.

## The Interrelationships of Variables in Video-Based Animal Studies—Scope for Conflict

**Figure 1:**
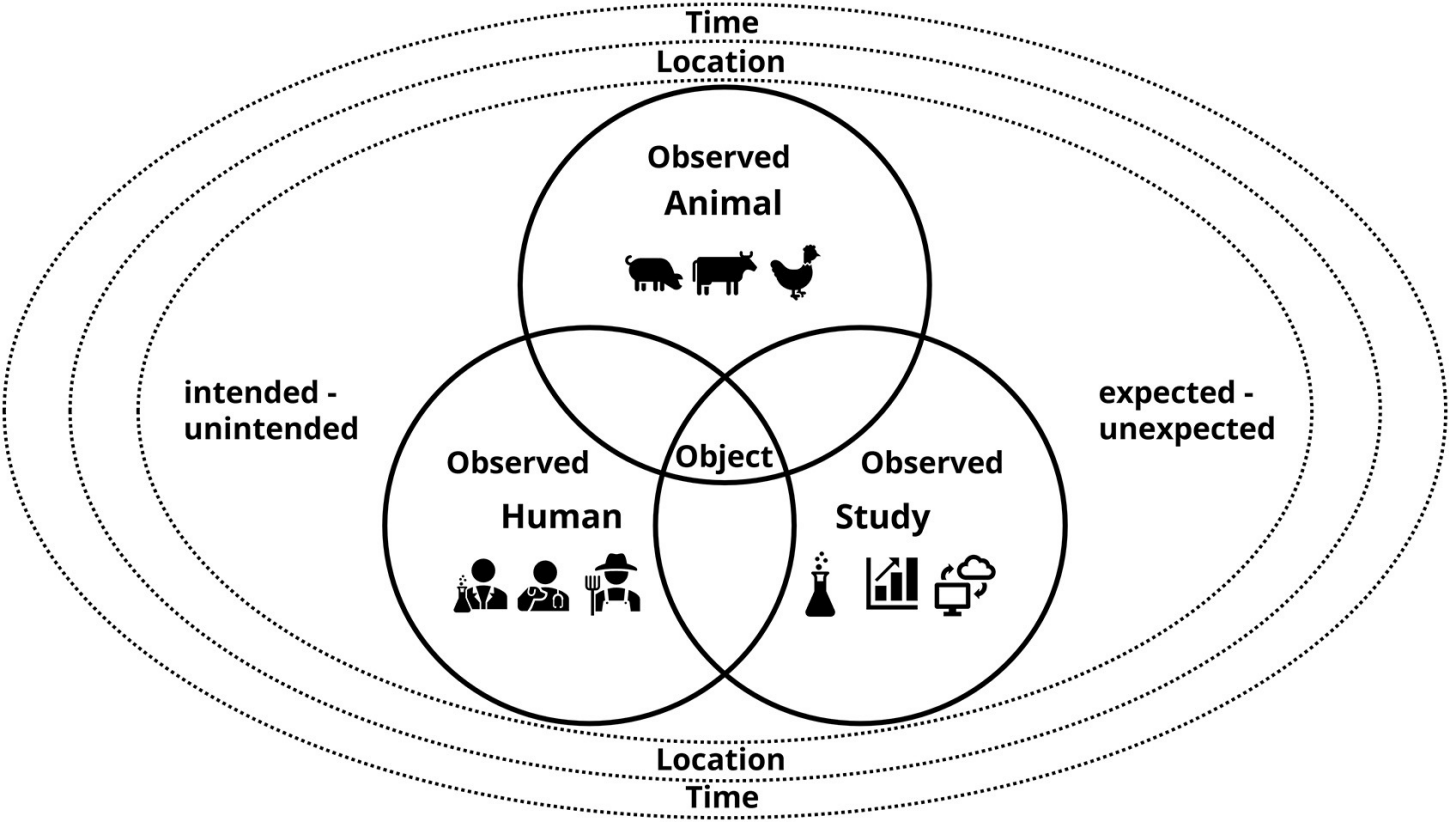
Variables the observer is confronted with in video-based animal studies: the object (i.e., what is seen on the video) and the observed: the study animals, humans in various roles and aspects of the study (e.g., technical equipment) captured on the video. The object and the observed can be on the continua of expected—unexpected and the intended—unintended. All variables are situated within the broader dimensions of location and time.

In order to map and better understand the conflicts that can occur during video analysis, it is important to be aware of the dimensions of the study on which the recordings are based. It is evident that three main variables are involved: the object or the event that is seen, the observed and the observer (Figure 1). The observed are typically the subjects of research, the animals, but also humans in various roles appearing on the footage, and any technical equipment, such as feeders, which are summarized under “study” here. Disturbances of one variable are likely to (but do not necessarily have to) affect the other variables. Leaving the lights on during night will have an impact on both the observed animals and the study, whereas providing slightly different enrichment materials may have no consequences for animal welfare, but will have an impact for the progress of the study. Defects of technical equipment as part of the study, such as skipping a feeding time with automatic feeders, are an example for disturbances which influence their own dimension (the study) and the animals. The event that is observed can be on the continua of being expected and unexpected, and on the one of being intended and unintended. The animals, for instance, are no passive factors of the study, they may show behaviors that are unexpected and not of interest for the research question. In a study assessing the use of a new perch for laying hens, feather pecking behavior may not be part of the ethogram, however, its accidental occurrence will affect animal welfare and the outcomes of the study. Furthermore, during maintenance work in the facility there could be an electricity failure, which is unintended, but which may lead to equipment not working properly and to consequences for the animals and the study.

While these interrelationships hold for research on animal behavior and welfare in general, video-based studies come with the additional challenges of the location- and the time-variable. Approaching the above mentioned situations may differ depending on whether the study is located on a commercial farm, in a shelter or at a research facility of a university. A further aspect of the location-variable in video-based studies is the discrepancy between the place where the recordings are made and the place where they are analyzed. In the simplest case, the recordings are made in the research facility and are evaluated in the observer's office at the same university. However, in international research projects it is common that videos are created in one country to be analyzed in another part of the world. Furthermore, video recording and analysis are situated within the dimension of time. Video analyses in animal behavior and welfare research are typically not performed in real time, but in retrospect, and the time between video production and analysis can vary considerably. Video observations may be carried out while the experiment is still running. However, at the time of video analysis the experiment has often already been completed, the batch of animals has already been slaughtered, or the people in charge have long since been working elsewhere.

Furthermore, video as a tool creates a tension between intensity and partiality of recorded situations. On the one hand, disturbances may be registered more likely and more frequently during video analysis than during live observations, where the researcher is usually only present in the facility for a limited period of time and does not have the possibility to fast-forward or rewind. On the other hand, the partiality, which is inherent in all kind of data (10), may be particularly obvious when the observer cannot follow the observed and cannot know what will happen outside the camera's field of view. It should be noted that this does not only apply to the video images, but also to the audio track, which in some cases is recorded along with the videos. Although maybe less frequent and less explicit than deviant behavior, unexpected vocalizations of the study animals could be heard during video analysis, indicating for instance a health or welfare problem. In the same way, ambiguous conversations of humans in the recording area could be overheard without having the possibility to ask directly what is meant by what is said. Thus, situations on video may be observed and possibly also overheard out of context.

Conflicts may arise when the observed disturbance is weighed against the original intention of the video recordings: the cameras have been installed for analyzing certain animal behaviors, not for surveilling the work of caretakers, monitoring technical processes or assessing the overall welfare status of the animals. In addition, those who are confronted with these situations first, the observers, are in most cases less experienced researchers, like students and PhD candidates. With a lack of experience, the conflicting interests between social expectations, animal health and welfare, and research quality and integrity, may cause uncertainty in the observers. This may lead to the observers finding themselves in a situation that they perceive as a dilemma. Dealing with such situations is largely dependent on the individual observers, their relationship with the principal investigator (PI), and the hierarchical structure and the culture of the research group. Revisiting the case of the caretaker mistreating the animals, one may argue that the severe consequences for the animals and the study should be a clear signal to take action for anybody observing such an event. However, a more junior observer may feel guilty about the consequences that such action can have for the caretaker, for instance that they will lose their job. The observer may also be worried about potential threats they might face from the person they observed and reported, particularly when the location and time of video analysis are close to video production. In addition, even more complex conflicts may occur when observing minor or more subtle disturbances, where the weighing of the interests at stake leads to less clear conclusions. Observers should be prevented from experiencing these situations as dilemmas, which overwhelm or paralyze them. Instead, they should be enabled to recognize such situations as conflicts, which may be complex, but amenable to intervention or even solution. To achieve this, and to guarantee high standards regarding privacy and research integrity, further reflection is needed within the animal behavior and welfare science community. The aim should be to establish guiding principles on how to approach conflicting situations during video analysis.

## Planning: the Principles of Privacy to Prevent Conflict

As argued above, a tension can occur between the observer's ambitions to preserve the privacy of the person recorded unintentionally and to disclose disturbances in the study. In order to prevent uncertainties and divergent understandings, the expectations and responsibilities regarding privacy should be made explicit during the early planning stage of the study, before the recordings are produced. A first step would be to clarify which principles of privacy play a role and how they relate to the specific context of video-based animal studies. In the social sciences, it is standard practice to obtain informed consent from the study participants to be video-recorded (5, 11, 12). Using video in this context often also requires strict reviewing procedures by institutional boards, similar to those needed for medical intervention studies to protect human research subjects (12). The reason for this is the non-anonymous nature of video. Anonymizing recordings is only possible by technically masking or filtering the participants' identities, which is laborious and would also render the data unsuitable for many research questions (12). The relative insensitivity of video to location and time can result in unpredictable sharing and re-using possibilities, involving unexpected and potentially also unintended future observers.

The question now arising is to which extent should humans involved in an animal study be treated as “research participants” in order to clarify and agree on the expectations and responsibilities regarding privacy? Treating them as research subjects would clearly miss the point, as the study is usually not intended to surveil their behavior. Nevertheless, humans may be unintentionally captured on video. Therefore, we propose to treat people, who are present at the location of the video recordings, such as researchers, caretakers or farmers, as research participants in a way that makes mutual expectations and responsibilities explicit. As a starting point, people need to be informed comprehensively about the nature and purpose of the footage being created. People do not only need to be aware that they may be recorded, but also that the videos will be watched. When planning a study, the PI should talk to the key personnel at the research location about video data collection, storage and anticipated place and time of analysis. This should be documented in a data management plan (DMP), which should be registered as metadata along with the videos. However, it cannot always be foreseen who will enter the location of the recordings. On a farm, for instance, an electrician may need to fix the lights at some point during the study. In this case, expectations and responsibilities could be made explicit by placing sheets on each entrance to the research location, which indicate the recording dates and times, and the contact details of the PI. In addition, key personnel should be encouraged to inform any person entering about the ongoing recordings.

In other words, arrangements should be made based on a shared and agreed moral frame of reference regarding privacy. However, in animal studies, a single focus on the prevention of privacy issues is not sufficient. As mentioned above, study animals are in a vulnerable position and depend on human care. The assumptions and responsibilities underlying such a duty of care or the expected human behavior toward animals should therefore be made explicit in a similar way as the privacy issues that may arise with video-based studies. A caretaker, for instance, needs to know which methods are state-of-the-art (and therefore expected on the recordings) for catching individual animals for health and welfare checks. Informing, educating or reminding people of these expectations and responsibilities during the planning stage of the study provides them with good conditions under which to choose their behavior. Under these “good conditions” one is likely to be responsive to the relevant reasons as described in Scanlon's value of choice account (13), i.e., the reasons that persons have for wanting things that happen to them, their obligations to others and vice versa, to depend on how *they* respond when faced with the alternatives. The value of choice is variable, and there may be situations in which a person would not want to choose or is worse-off by having this opportunity (13). However, in a video-based animal study, it can be assumed that people would value to know when and where they may be recorded and what will happen with these recordings. This will also apply to the observer: knowing the observed had the opportunity of choice under good conditions, will eventually address part of their uncertainty of how to deal with conflicting situations during video analysis. What matters in determining a person's responsibility for an outcome is the opportunity of choice under good conditions, not whether a conscious choice is actually made. Therefore, prior to the analysis, the DMP and the arrangements made therein should be outlined to the observer. They will get an overview of the context and purpose of the videos. They will also notice that it was ensured that unintentionally recorded persons were aware of being filmed and the videos being analyzed. These considerations may be complicated by the location- and particularly the time dimension. Not all persons involved in the study will be so right from the planning stage. However, creating a shared frame of reference could go beyond bargaining with actual contractors. When actual bargain is not possible, acting in justifying oneself to others may be an option. The question the PI should ask is whether persons involved at a later stage could reasonably reject a principle permitting an action, or whether they could reasonably object the arrangements made in the DMP (14).

However, these considerations are only useful if the unexpected or unintended observed are persons, with whom one can actually or hypothetically create and share a frame of reference regarding privacy and a duty of care toward animals. The animals showing unexpected behaviors when the study is already running or the automatic feeder skipping a feeding time, which is only noticed during retrospective video analysis, call for a different approach.

## Running and Reporting: the Principles of Research Integrity to Create a Culture of Openness

Unintentionally recorded and unexpected animal behaviors or technical defects during the study, which may in addition be minor and temporary, can easily end in issues of research integrity (RI). RI can be defined as a matter of careful and precise work that meets the methodological standards of the relevant field, and transparency in reporting and justified interpretation of all findings (6). Particularly the last two aspects are at stake when the weighing of the observed object against the intention of the research or other expectations leads to a perceived dilemma, and the observer fails to handle the situation or to act at all. Again, the caretaker recorded while mistreating the animals may be a rare occasion, similar to serious violations of RI, such as falsification, fabrication and plagiarism (15). However, failing to notice, document and report a relevant observed object can be a case of questionable research practices (15). Such objects, the automatic feeders skipping a feeding time or the animals engaging in deviant behaviors, impact the conclusions which can be drawn from the study and in how far they can be generalized and reproduced. It may be argued that any impairment of the study animals' welfare will affect the study results and will thus provide scope for RI issues if not properly documented and reported. However, an interesting question for further research would be whether safeguarding animal welfare could become a principle of RI on its own, regardless its instrumental value for the quality of the research outcomes.

Without delving deeper into the principles of RI here, it seems appropriate to tackle the problems described above by measures designed to promote RI: creating a culture of openness which enables the discussion of noticed but unexpected or unintended disturbances during the study. Recently, many research organizations worldwide have adopted and customized the more general declarations and codes of conduct for RI (16). However, translation into practice and to specific situations remains difficult. Education is one way to promote RI and to relate its principles to specific cases. Pizzolato et al. (17) published an inventory of more than 200 freely available online RI educational resources. The majority of these resources are not customized based on scientific domain, and deal with more general topics, such as research misconduct, publication ethics and authorship-related issues, and data management (17). Tools that address RI related issues in video-based studies are not mentioned.

The first step to mitigate the risk of RI issues is to create awareness of the unexpected and the unintended that may be observed. When instructing the observer, the PI should not only make sure that the research question and the ethogram are understood, but also inform about possible disturbances that may be seen. Second, open and active reporting of such events should be encouraged, for instance by adding a column for “other behaviors observed”, or “general remarks” to the score sheets. The challenge here is to not create a compliance-based approach including a set of rules and checklists, which in the worst case may give a legalistic impression (18). Such a framework would be viewed as burdensome or may increase the uncertainty of the observer, who might fear of being controlled and prosecuted. Instead, the observer should experience that adhering to the principles of RI is not something imposed from outside, but something arising from the purpose of science itself (18). Therefore, Pennock and O'Rourke (18) proposed a virtue-based approach to RI, i.e., to integrate the scientific virtues, the qualities embodied by the exemplary scientist, into RI and science ethics training. Without going further into the training methods here, the approach seems applicable to the specific case of video-based animal studies. How would noticing but ignoring an omitted feeding time fit with the core scientific virtues of curiosity and honesty? Would this behavior contrast the central aim of science practice, i.e., the discovery of empirical truth about the natural world, and its methods' basic epistemic values of testability and repeatability (19)?

Implementing such an approach can only be successful if a culture of openness is created for discussion and reflection on the conflicts the observer may encounter. These discussions should also leave room for the conclusion that—when viewed with more developed practiced dispositions—the disturbance recognized by the observer is no conflict at all and needs no further consideration. However, it may be more often that action is needed, and depending on what is observed there may be various possibilities. In this case, it is also important to adhere to the previously agreed provisions regarding privacy. The content of an unintentionally recorded private conversation between caretakers in an animal facility needs to stay private. However, if their talk, for instance due to its volume, may affect the animals or the study, “loud talk” needs to be reported without referring to the content. There needs to be clarity on whom to consult when conflicts are noticed. For the observer this should be the PI in the first instance, but the discussion should not end at this level. The event could for instance be re-watched with experts or other persons involved in the study to come to more nuanced conclusions; measures could be taken to alleviate deviant animal behavior (if the experiment is still running), or further data analysis and interpretation of results could be adjusted. A good example for the latter option is the study by Cronin et al. (20), even though it is not video-based. The authors found that an unexpected and unintended rainfall event contributed to the development of feather pecking and cannibalism in laying hens and overshadowed the experimental treatments (provision of foraging material and exposure to stressors). The researchers noticed the unexpected event and its unintended consequences and came to the conclusion that it would not be an option to continue with the intended analyses. Instead of perceiving this as a dilemma, they chose to adapt the interpretation and publish the unexpected findings. Despite lack of generalization, the results of the study are still valuable for designing future experiments, and the way they are reported is in line with the values of testability and repeatability.

## Discussion

In this article we aimed to raise awareness of the interrelationship of the object, the observed and the observer in the context of video-based animal behavior and welfare studies, and the potential conflicts that may emerge from these special relations. To deal with the conflicting interests between social expectations, animal health and welfare, and research quality in practice, we propose stepping stones to a framework which translates the basic concepts of privacy and research integrity to the specific case of this type of video-based research.

The suggestions outlined above can be summarized by two practical implications: (a) fostering systematic reflection on the interests at stake, such as privacy of persons involved, animal welfare, and research integrity, and (b) applying a participatory approach when dealing with these interests. Systematic reflection presupposes awareness of the variables and dimensions involved in the study, their interrelationship, and the conflicts that may arise from them. In addition, systematic refection needs to begin at the early planning stages of the study, and to continue through data collection, analyses and publication of results. This also applies to the implementation of a participatory approach. A participatory approach starts with making actual or hypothetical “contracts” with people informing them about the nature, purpose and intended analysis of the recordings based on a shared moral frame of reference. However, participation does not end at this point: videos are sharable and people involved in the study—other than the observer—can be invited to watch certain sequences (10). These may for instance be situations in which they were captured unintentionally on the video, or events of deviant animal behavior or malfunction of technical equipment, which are difficult to interpret due to the partiality of the recordings. It is important that people do not get the impression that the purpose of re-watching is to criticize or prosecute their behavior in the video, but that their interpretation—as participants in the research project—is asked for in order to gain a more diverse view on potentially ambiguous events. A culture of openness can decrease the gap between researcher and animal owner or farmer and may help to foster collaboration and co-create ethical practice in video-based animal studies.

As mentioned before, this approach may in part be limited by the location- and time dimension of video-based studies: not all people in charge of the animals during the experiment will be available for revisiting certain situations on video during the time of analysis. However, these potential hurdles may only emphasize the mediating role and the responsibility of the PI. The PI is the one who has an overview over the whole study and the people involved in its different stages. This entails more than only an attitude of careful reflection by the PI. It also requires that they encourage this process in the observer and other persons involved by raising awareness of potential conflicts, making them discussible and offering opportunities on how to deal with them, i.e., creating a culture of care and openness. This culture of care and openness should also provide clarity on how the observer will be protected in case they observe and report unlawful events, such as the mistreatment of the study animals. To support them in this task, educational materials based on the proposed framework should be developed, such as small videoclips, charts or factsheets, which are targeted at the observer, but also at people working at the location of the experiment. However, to prevent handing out checklists or decision trees, which may at worst lead to more uncertainty in the observer, the approach should be outlined and guided by conversation. A further limitation may be that on the one hand PIs might not always be aware of the potential conflicts arising during video-based studies themselves, or of the dilemmas the observer may face. As the PI may have already practiced the virtues of science, for them, it would be self-evident to document and report disturbances observed on video, whereas for a more junior observer this might not be the case. On the other hand, we are aware that some research groups engaging in video-based animal behavior and welfare studies will already have established the concepts of systematic reflection and participation in their daily work. However, we aim to raise awareness where this is not the case and offer a systematic approach where refinement of daily practices is necessary. The recent discovery that many published scientific results do not withstand reproduction by independent labs culminated in the “reproducibility crisis” in various disciplines (21), from which the animal sciences are no exception (22). This issue is mainly attributed to poor study design, the use of inappropriate statistical methods, and extreme standardization within experiments (21, 22). However, Bailoo et al. (23) found that systematic variation of factors in an animal experiment lead to effects smaller than expected. The authors concluded that other aspects that vary among labs may be accountable for the observed differences between replicate studies, and that further research is needed to identify the source of variation in the results (23). This gives reason to assume that part of the “reproducibility crisis” might be also due to unnoticed, undocumented and unreported disturbances occurring on video recordings.

Recent developments in the field of animal behavior and welfare research show a shift toward methods of automated video analyses that do not involve human observers (24). However, developing these methods usually requires annotations of a large number of video frames by humans (25, 26). During the annotation process, similar unexpected or unintended events could be observed as during manual video observation. In addition, even with fully established automated video analyses, the generated data could be indicative of disturbances during recording, which would make it necessary to watch the raw footage. This, in turn could then lead to the conflicts outlined above.

To conclude, only by making expectations and responsibilities regarding privacy and human behavior toward study animals explicit, creating a culture of openness as a precondition, raising awareness of potential conflicts, and practicing systematic reflection and co-creation, can researchers conduct video-based animal studies, which ensure high levels of animal welfare, research quality and integrity, while at the same time preserving the privacy of all persons involved.

## Author Contributions

MG and FM developed the concept and prepared the manuscript. Both authors read, edited and approved the final manuscript.

## Conflict of Interest

The authors declare that the research was conducted in the absence of any commercial or financial relationships that could be construed as a potential conflict of interest.

## Publisher's Note

All claims expressed in this article are solely those of the authors and do not necessarily represent those of their affiliated organizations, or those of the publisher, the editors and the reviewers. Any product that may be evaluated in this article, or claim that may be made by its manufacturer, is not guaranteed or endorsed by the publisher.
